# Physicochemical Properties, Bioactive Components and Volatile Compounds of Dietary Fatty Acid Balanced Blend Oil

**DOI:** 10.3390/foods15111840

**Published:** 2026-05-22

**Authors:** Enhui Liu, Qing Niu, Liangliang Lu, Lingxin Geng, Jie Yang, Huawen Yao, Zhongkai Zhao

**Affiliations:** 1 College of Smart Agriculture (Research Institute), Xinjiang University, Urumqi 830017, China; liuenhui_nori@126.com (E.L.);; 2 Xinjiang Key Laboratory of Biological Resources Genetic Engineering, College of Life Science and Technology, Xinjiang University, Urumqi 830017, China; 3 Xinjiang Walnut Industry Engineering Technology Research Center, Xinjiang Zhejiang Fruit Industry Co., Ltd., Aksu 843000, China

**Keywords:** blended oils, fatty acid balance, physicochemical properties, aroma compound profiles

## Abstract

Walnut oil is growing in consumer demand due to its rich nutritional profile; however, its fatty acid composition exhibits an imbalanced SFA:MUFA:PUFA ratio (0.13:0.18:1). To improve the fatty acid balance using locally available vegetable oils in Xinjiang, we investigated the effects of blending walnut oil with linseed oil, safflower seed oil, sunflower seed oil, rapeseed oil, peanut oil, and soybean oil on physicochemical indexes, fatty acid composition, and bioactive components. Aroma characteristics were assessed by E-nose and HS-GC-IMS. The results showed that the acid value and peroxide value of the blended oil decreased, while the content of vitamin E and squalene increased inversely. The ratio of ω-6/ω-3 maintain steadily at 4–6:1, and the ratios of SFA, MUFA, and PUFA were close to 0.27:1:1. Significant differences were observed between the aroma characteristics of walnut oil and the blended oil. HS-GC-IMS identified 85 volatile organic compounds (VOCs), among which walnut oil had a higher content of alcohols, aldehydes, and ketones, with 4-hydroxy-5-ethyl-2-methyl-3(2H)-furanone as its characteristic aroma compound. The acetophenone serves as the key aroma component after blending, and the unique flavor components of each base oil (e.g., 4-nonanone in linseed oil, 3-methyl-2-pentanone in rapeseed oil, etc.) exert a synergistic effect after rationing to present a composite aroma characteristic of blended oils, which mainly consists of 3-methylbutyl butyrate and 4-ethylphenol.

## 1. Introduction

Dietary fat is one of the three most important energy-providing nutrients required by the human body, and it is crucial to reasonably control the intake of fat in daily dietary planning. In fact, a reasonable ratio of fatty acids is essential for maintaining normal physiological functions of the human body and preventing chronic diseases. The World Health Organization (WHO) considers the antioxidant capacity, the ratio of saturated, monounsaturated, polyunsaturated fatty acids, and essential fatty acids as important factors in evaluating dietary fats and oils [1]. Vegetable oil dominates the edible oil market, dietary oil intake not only supplying energy but also providing fatty acids needed for human growth and metabolism, while promoting the absorption of fat-soluble vitamins. Vegetable oil also contains tocopherols, polyphenols, phytosterols, squalene and other micronutrients.

Walnut oil is mainly composed of fatty acids such as linoleic acid and α-linolenic acid, with essential fatty acids comprising up to 70% of its mass. Research indicates that walnut oil may serve as a dietary adjuvant for type II patients with hyperlipidemia [2]. Moreover, its high polyunsaturated fatty acids (PUFA) content supports cardiovascular health by lowering blood pressure and LDL cholesterol levels [3]. Apart from walnut oil, other special oils in Xinjiang also have their own dietary characteristics. Flaxseed is a rich source of α-linolenic acid (ALA), an essential ω-3 fatty acid critical for human health, which supports brain development, prevents cardiovascular disease, regulates blood lipid, and inhibits tumor growth [4]. Safflower seed oil, abundant in vitamin E and phytosterols, demonstrates efficacy in modulating cholesterol and glucose homeostasis [5]. Sunflower ranks among the world’s leading oilseed crops, alongside rapeseed, soybean, and cottonseed, in terms of production volume [6]. Oleic acid, which is abundant in canola oil, is a monounsaturated fatty acid (MUFA) that is more stable than other unsaturated fatty acids and has been linked to lower blood cholesterol and cardiovascular protection [7]. Soybean oil, predominantly composed of linoleic acid and α-linolenic acid, contains vitamin E and vitamin K and other biologically active components that protect cellular membranes from free radical damage, mitigate oxidative stress-induced cellular injury, and prevent chronic diseases [8]. Peanut oil is rich in linoleic acid (18:2, an omega-6 polyunsaturated fatty acid). The total content of unsaturated fatty acids is about 80%. Its fatty acid distribution is similar to that of olive oil, which may reduce the risk of cardiovascular disease [9].

Single vegetable oils do not fulfil the requirements for fatty acid balance, nutritional quality and organoleptic properties, and adequate oxidative stability. Blending different vegetable oils is a practical way to produce new specific products with desirable structural and oxidative properties. Blending vegetable oils with different physicochemical properties may bring up various functional properties, as well as improve their applications in food [10]. For example, adding rapeseed oil to refined palm oil concentrate and virgin olive oil, the blended oils have shown higher thermo-oxidative stability [11].

Apart from the processing attributes, blending vegetable oils can optimize balanced fatty acid intake, thereby reducing obesity indices, improving blood lipids, and mitigating cardiovascular risks, which helps prevent obesity and metabolic diseases [12]. Studies have shown that the mixture of sesame oil and rice bran oil has hypotensive and hypolipidemic effects, showing synergistic benefits with antihypertensive drugs [13]. At the same time, blended oils can balance the inherent properties of individual oils to achieve a more desirable organoleptic profile [14].

The aim of this study was to formulate balanced oil blends aligned with the optimal ω-6/ω-3 ratio (4–6:1) and SFA: MUFA: PUFA ratio (0.27:1:1), focusing on Xinjiang’s oilseed resources, and to characterize their physicochemical properties, fatty acid compositions, bioactive components, and volatile profiles.

## 2. Materials and Methods

### 2.1. Materials

Shelled walnut kernels and stripped flaxseed kernels were purchased from Alashankou Jinmu Technology Co., Ltd. (Urumqi, China); sunflower seeds, safflower seeds, soybeans, peanuts and rapeseeds were purchased from Xinbeiyuan Spring Market in Urumqi (Urumqi, China). Briefly, 37-component fatty acid methyl ester mixed, Wechsler’s reagent, vitamin E standard, squalene standard and β-sitosterol standard (purity ≥ 99%) were purchased from Shanghai Yuanye Bio-Technology Co., Ltd. (Shanghai, China). All other chemicals and reagents were of analytical grade.

### 2.2. Preparation of Vegetable Oil

Firstly, walnut kernel, linseed, sunflower seed, safflower seed, rapeseed, peanut and soyabean were sieved to remove the shells and impurities, and walnut kernel and peanut kernel were chopped to the size of soyabean kernel. The vegetable oils were extracted using a ZYJ-420 type screw oil press at a temperature of 120–130 °C and a pressure of 5–8 MPa. Subsequently, the seven raw oils were centrifuged at 8000 rpm for 20 min. The clarified vegetable oils were placed in glass bottles of oil and stored at −25 °C protected from light for subsequent analysis.

Walnut oil, linseed oil, safflower seed oil, sunflower seed oil, rapeseed oil, soya bean oil and peanut oil were blended in different mass percentages with the blending objectives of SFA:MUFA:PUFA = 0.27:1:1, and ω-6/ω3 PUFA = 4–6:1. Table 1 shows five different combinations of blended oils in different percentages. Following the method of Chang et al. [15], each blended oil was stirred at room temperature with a thermostatic magnetic stirrer (ZNCl-BS, Beijing, China) at 300 rpm/min for 20 min, then stored at 4 °C for use.

### 2.3. Physicochemical Analysis

#### 2.3.1. Iodine Number

The iodine number was determined according to GBT 5532-2022 (2022) [16].

#### 2.3.2. Acid Value

The acid value was determined according to GB 5009.229-2016 (2016) [17].

#### 2.3.3. Peroxide Value

The peroxide value was determined according to GB 5009.227-2023 (2023) [18].

#### 2.3.4. Saponification Number

The saponification number was determined according to GB/T 5534-2008 (2008) [19].

### 2.4. Determination of Fatty Acid Composition

The fatty acid composition of vegetable oil samples was analyzed by GC-MS (7890B-7000D, Agilent Technologies, Shanghai, China) [14]. Fatty acid methyl ester was prepared as follows: firstly, 1.0 mL of 0.5 M sodium methoxide in methanol was added to the oil sample, followed by shaking well for 10 min, then 1.0 mL of n-hexane was added by vortexing for 2.0 min; after that, the solution was mixed with saturated NaCl solution. The mixture was allowed to phase-separate, and the upper n-hexane layer was aspirated and filtered through a 0.22 μm membrane into the GC-MS analysis.

Gas chromatographic conditions: SP-2560 capillary column (100 m × 0.25 mm × 0.20 μm) was applied, the initial temperature of the column was set at 140 °C for 5 min, then the temperature was increased to 200 °C at 10 °C/min and kept for 30 min, and then consistently increased to 240 °C at 4 °C/min, and kept another 19 min, finally with a total time program control of 70 min. Carrier gas: high-purity helium, column flow rate of 0.7 mL/min, inlet temperature of 270 °C. Mass spectrometry conditions: inlet temperature 280 °C, ion source temperature 230 °C, electron bombardment ionization (EI) source, SIM scan mode, electron energy 70 eV.

### 2.5. Determination of Vitamin E, β-Sitosterol, and Squalene Content

Vitamin E, squalene and sterols were analyzed using a high-performance liquid chromatography (HPLC) system (Agilent 1260, DAD + FLD detector, Agilent Technologies, Shanghai, China) [20]. 5.0 g of sample was accurately weighed into a 250.0 mL conical flask, then 50.0 mL of 1.0 M potassium hydroxide-ethanol solution was added, and the mixture was refluxed in a 90 °C water bath for 2.0 h. The saponified solution was extracted by adding hexane, and the extract was washed with 10% ethanol after 3–4 extractions. The solvent was removed by rotary evaporator, and the residue was fixed in a 5.0 mL volumetric flask with methanol/dichloromethane (*v*/*v* = 1:1) solution, filtered through a microporous filter membrane (0.45 μm), and analyzed for the bioactive components by normal-phase HPLC. Liquid chromatography conditions: Detection wavelength 202 nm, C18 reversed-phase column (250 mm × 4.6 mm, 5 μm), mobile phase methanol, flow rate 1.0 mL/min, column temperature 30 °C. Standard curves were constructed by plotting peak area (Y) against standard concentration (X) (Figure S1). The results showed good linearity between the content of vitamin E, squalene, and β-sitosterol and their peak areas within the range of 0.1 mg/mL. The linear regression equations were: Y = 8180.2x + 11.709 (R^2^ = 0.9999); Y = 3074.5x − 9.1617 (R^2^ = 0.9989); Y = 21,483x − 7.5657 (R^2^ = 0.9996).

### 2.6. Volatile Analysis

#### 2.6.1. Analysis of Electronic Nose

Oil samples (3.00 ± 0.01 g) were weighed into a 20 mL headspace vial sealed with a PTFE spacer. Vials were then incubated in a water bath at 60 °C for 10 min. The headspace was injected with an electronic nose (C-PEN3, Penotec, Beijing, China) tip at the same height. The parameters were set as follows: cleaning time of 120 s, pre-injection time of 5 s, injection flow rate of 400 mL/min, carrier gas flow rate of 400 mL/min, and detection time of 70 s. Three sets of test oil samples were made in parallel. The sensor signals at the stable plateau time point (52–56 s) were taken for data analysis [21].

#### 2.6.2. Gas Chromatography-Ion Mobility Spectrometry (GC–IMS)

Volatile flavor components of various vegetable oils and their blended oil products were characterized using a Gas Chromatography-Ion Mobility Spectrometry (GC–IMS) (FlavourSpec^®^, Gesellschaft für Analytische Sensorsysteme mbH, Dortmund, Germany) [22]. Firstly, 3.0 g of the oil was transferred to a 20.0 mL headspace vial, incubated at 70 °C for 10 min, and then injected into the instrument. The injection port temperature was 75 °C, with a pre-injection wash time of 30 s and a post-injection wash time of 6 min. The GC conditions were as follows: FS-SE-54-CB-0.5 quartz capillary column (15 m × 0.53 mm × 0.5 μm); column temperature held at 60 °C; and nitrogen as the carrier gas. The carrier gas flow rate was 2.0 mL/min for the first 2 min; 2–5 min, from 2 mL/min to 15 mL/min; 5–10 min, from 15 mL/min to 50 mL/min; 10–15 min, from 50 mL/min to 100 mL/min; 15–20 min, from 100 mL/min to 150 mL/min. The volatile organic compounds (VOCs) in edible vegetable oils were identified by comparing the retention indices and the drift times of the standards in the GC-IMS library using GC×IMS Library Search software (v 1.0.3). The test oil samples were made in three parallel groups.

#### 2.6.3. Calculation of ROAV

Odor Activity Value (OAV) and Relative Odor Activity Value (ROAV) are analyzed to obtain the contribution of each compound to the overall flavor. The OAV is commonly used to assess the contribution of aroma compounds; an OAV > 1 is considered to make a significant contribution to aroma characteristics Liu [23]. The volatile compound with the highest overall contribution to the sample (i.e., the one with the highest odor activity value) is selected as the standard, and its ROAV is 100, while the ROAV of the remaining volatile compounds is calculated according to the following formula [24]:

$${\mathrm{ROAV}}_{a}\;=\;C_{a}/T_{a}\;\times \;T_{\max}{/C}_{\max}\;\times \;100$$

where in C_a_ is the relative percentage of the remaining volatile compounds (%); C_max_ is the relative percentage corresponding to the volatile compound that contributes most to the aroma of the sample (%); T_a_ is the odor threshold of the remaining volatile compounds; and T_max_ is the odor threshold corresponding to the volatile compound that contributes most to the aroma of the sample.

### 2.7. Statistical Analysis

Laboratory Analytical Viewer (LAV) analysis software was used to view the spectrograms; the built-in NIST database and IMS database of GC-IMS Library Search were applied to characteristic two-dimensional signal peaks; the Reporter plug-in was used to view the difference plots; the Gallery Plot plug-in was used to construct the fingerprints of volatile compounds and analyze the differences. Origin 2024 (Northampton, MA, USA) was used for radar plotting, principal component (PCA), and other graphical analyses; SIMCA 14.1 (Sweden) was used for partial orthogonal least squares-discriminant analysis (PLS-DA) analysis. The peak area normalization method was used to obtain the relative volatile compound.

For comparisons among multiple groups, one-way analysis of variance (ANOVA) was performed, followed by Tukey’s post hoc test for pairwise comparisons. A *p*-value < 0.05 was considered statistically significant. All statistical analyses were performed using SPSS 27.0 (IBM Corporation, New York, NY, USA). All experiments were performed in triplicate and expressed as means ± SD.

## 3. Results and Discussion

### 3.1. Physicochemical Properties of Oils

Iodine value (IV), peroxide value (POV), and acid value (AV) are usually considered as the main significant indicators for assessing the quality and stability of oils [25]. IV is an important indicator for evaluating the degree of unsaturation of triacylglycerols; a higher IV indicates a greater degree of unsaturation of the oil [26]. In the present study, the IVs of the seven vegetable oils and blended oils were 87.63 (peanut oil) ~185.96 (safflower seed oil) g/100 g with significant differences (*p* < 0.05). Among them, the IV of the five blended oils was lower than that of walnut oil, which was mainly attributed to the reduction in PUFA content. AV was related to the content of free fatty acids (FFA) in vegetable oils, which are subjected to hydrolysis by environmental factors, thereby producing FFA, leading to the oxidation of the vegetable oils [27]. The AVs of safflower seed oil (1.5 mg/g) and rapeseed oil (0.93 mg/g) were higher, and the AVs of five blended oils were lower than 0.80 mg/g. The POV of the seven vegetable oils and five blended oils ranged from 2.00 to 13.92 meq/kg and from 2.89 to 8.91 meq/kg, respectively. Among them, soybean oil and linseed oil exhibited relatively high POV, which may be attributed to their high contents of linoleic acid and α-linolenic acid. These polyunsaturated fatty acids are highly susceptible to oxidation induced by environmental factors such as light, temperature, and oxygen. The AV and POV of vegetable oils and blended oils were below both the maximum upper limits specified in the Chinese National Food Safety Standard for vegetable oils (AV ≤ 3.0 mg/g; POV ≤ 0.25 g/100 g, which is equivalent to 19.7 meq/kg) and the maximum permissible limits stipulated by the Codex Alimentarius (AV ≤ 4.0 mg/g; for virgin oils, POV ≤ 15 meq/kg) [28,29].

### 3.2. Nutrient Content

Vitamin E (VE), as an essential fat-soluble nutrient, helps neutralize free radicals and provides protection against oxidative stress, thus playing a vital role in the prevention of a wide range of diseases and in the maintenance of overall health [30]. The VE content of the seven vegetable oils ranged from 1.86 to 73.14 mg/100 g, with sunflower oil and safflower seed oil having higher VE content. The VE content of the five blended oils was significantly higher than that of walnut oil, and among the blended oils, B-5 had the highest VE content (3.93 mg/100 g).

Phytosterols can be defined as secondary plant metabolites belonging to the triterpene family, with a tetracyclic ring and a side chain linked to a central carbon structure, and are endogenous to all plant-derived food ingredients [31]. Among them, β-sitosterol is one of the major phytosterols detected in vegetable oils, Singh et al. [32]. found that high levels of sterols significantly reduced the formation of glycerol trioleate and triacylglycerol polymers in vegetable oil samples, suggesting that sterols exhibit an antioxidant effect at frying temperatures. The β-sitosterol content of the seven vegetable oils ranged from 98.01 to 401.50 mg/100 g, while that of the five blended oils was significantly reduced.

Squalene is an intermediate hydrocarbon in the biosynthesis of phytosterols and terpenes. The squalene content of the seven vegetable oils ranged from 0.89 (walnut oil) to 37.21 mg/100 g (peanut oil), with peanut oil’s squalene content being significantly higher than that of the other vegetable oils. The squalene content of the five blends showed an overall increasing trend compared to walnut oil, mainly attributed to the increase in peanut oil content.

### 3.3. Fatty Acid Composition of Oils

The fatty acid compositions of the seven vegetable oils are listed in Table 2. A total of 13 fatty acids were detected in the seven vegetable oils, mainly including oleic, linoleic, and α-linolenic acids, with small amounts of saturated fatty acids (e.g., palmitic and stearic acids). Walnut oil, sunflower oil, safflower seed oil and soya bean oil contained high levels of linoleic acid; the MUFA content of rapeseed oil (61.17%) was significantly higher than that of other oil varieties, mainly because of the highest content of oleic acid (61.05%) in rapeseed oil. In addition to this, peanut oil was also rich in oleic acid, with a high level of 41.34%, and linseed oil contained a high level of α-linolenic acid, with a high content of 54.72%.

The right ratio of fatty acids helps to maintain the normal physiological functions of the body, and the Dietary Guidelines for Chinese People suggest an optimal ω-6:ω-3 ratio of 4–6:1 [33]. Zhou et al. [34] reported that an SFA:PUFA:MUFA ratio of 0.27:1:1 may reduce serum total cholesterol and LDL cholesterol. Walnut oil had an ω-6/ω-3 ratio of 4.99 but was low in monounsaturated fatty acids; sunflower oil, safflower seed oil, and soya bean oil had over 50% omega-6, resulting in an out-of-range omega-6/3 ratio. Excessive intake of ω-6 fatty acids may interfere with renal metabolism and promote inflammation linked to heart disease, among other diseases [35].

After blending the seven vegetable oils, the ω-6:ω-3 ratios in the prepared blended oils ranged from 4.53 to 4.67, and all SFA:MUFA:PUFA ratios approached 0.27:1:1, indicating that the five blended oils fulfilled the proportioning requirements (Table 3). The MUFA content of the blended oils increased through the addition of linseed oil and peanut oil. Among them, the linoleic acid content of B-1 oil (36.32%) was significantly higher than that of the other four blended oils. Gu et al. [36]. found that blends of linoleic acid-rich soybean oil, ω-9-rich rapeseed oil, and peony seed oil inhibited α-amylase and α-glucosidase, revealing their potential as hypoglycemic functional foods. It can be seen that mixed vegetable oil is an economical way to change its fatty acid composition and physicochemical properties [10].

### 3.4. E-Nose Analysis

Electronic noses are widely used for the analysis of volatile compounds in food products by mimicking the sense of smell and have a more accurate way of characterizing the aroma profile of food products than the human nose [37].

**Figure 1:**
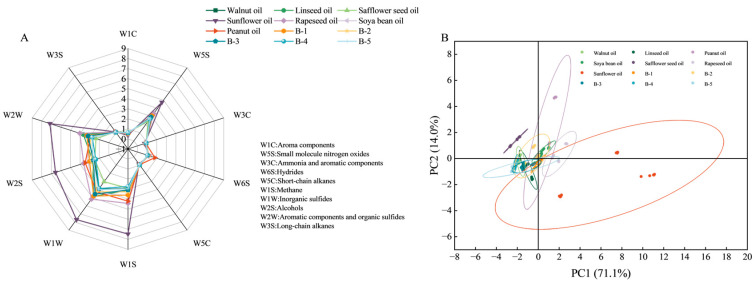
Radar (**A**) and PCA (**B**) of vegetable oils and blend oils detected by electronic nose.

Radar plots of sensor response values for seven vegetable oils and blended oil were plotted based on 10 sensors with differential response values for volatile components of different vegetable oils (Figure 1A). Seven vegetable oils had similar odor profiles with higher response values for W5S, W1S, W1W, W2S and W2W, with sunflower oil having higher response values than all the other vegetable oils, indicating that the concentration of volatile constituents, such as methyl groups, aromatic compounds, and organic sulfide. Blended oils showed sensor responses closer to walnut oil, peanut oil, and rapeseed oil. It is necessary to characterize the volatile organic components in the blended oils by the GC-IMS technique.

The results of the PCA plots were shown in Figure 1B, with PC1 and PC2 contributing 71.1% and 14.0%, respectively, for a total contribution of 85.1%. Walnut oil, linseed oil and soya bean oil had the most concentrated data points, while sunflower oil had the highest dispersion, and peanut oil was the next highest, indicating that walnut oil, linseed oil and soya bean oil were close to each other in terms of their own overall flavor profiles, whereas sunflower oil had a greater sample variation on its own. Notably, blended oils partially overlapped with walnut oil, rapeseed oil, and sunflower oil, suggesting that the overall flavor profile of vegetable oils can be altered with the mixing of vegetable oils with different mass percentages. To further explore the differences between vegetable oils and blended oils, their volatile compounds were comprehensively analyzed using HS-GC-IMS.

### 3.5. GC-IMS Analysis

#### 3.5.1. Identification of VOCs by HS-GC-IMS

**Figure 2:**
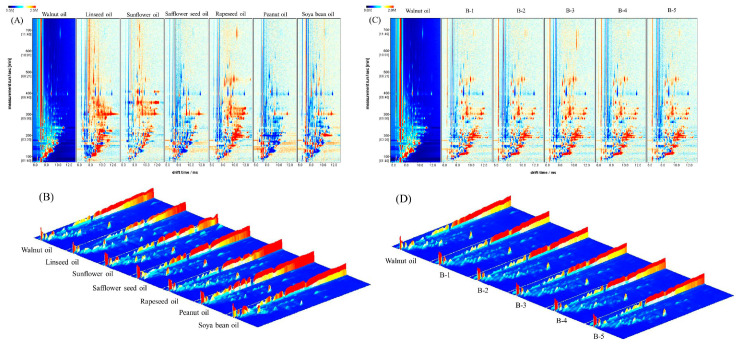
GC-IMS three-dimensional chromatogram of volatile components in vegetable oil samples (**A**); GC-IMS difference spectra (**B**) of volatile components in vegetable oil samples; GC-IMS three-dimensional chromatograms (**C**) of volatile components in blend oil samples; GC-IMS difference spectra of volatile components in blended oil samples (**D**).

GC-IMS can separate and identify different compounds by their different migration speeds in the electric field, which has the advantages of high sensitivity, simple operation and strong separation capability [38]. The data are represented by a three-dimensional topographic map (Figure 2B,D), where the *X*-axis represents the migration time of the reactive ion peak (Dt), the *Y*-axis represents the gas-phase retention time (Rt), and the *Z*-axis represents the intensity of the ion response. By normalizing the ion migration time and the reaction ion peak (RIP), the top view of the GC-IMS topographic map is shown in Supplementary Figure S1, where each point on both sides of the RIP represents a volatile organic substance, and the red and blue colors represent the higher and lower concentrations of the substances, respectively. There are differences in the VOCs among different vegetable oil samples, and in order to compare these differences more clearly, the spectrum of walnut oil was selected as a reference; the VOCs are shown as white when consistent with the reference, while red represents that the concentration of the substance is higher than that of the reference, and blue represents that the concentration of the substance is lower than that of the reference. A total of 85 volatile compounds were characterized from the different vegetable oils, including 19 esters, 18 ketones, 13 aldehydes, 14 alcohols, 7 acids, 4 phenols and 10 other compounds, and detailed information on all volatile compounds is given in Table S2. It is worth noting that p-methylacetophenone-M exists in two forms, monomer and dimer, and that dimer formation can be attributed to two molecules sharing a proton in the ionization region when compounds with high proton affinity are present in large concentrations [39].

**Figure 3:**
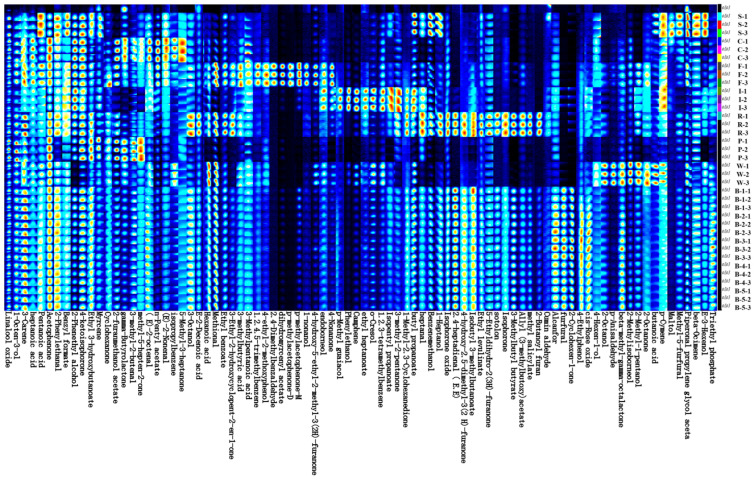
Fingerprint of volatile flavor substances in vegetable oils. Walnut oil (W), linseed oil (I), sunflower oil (F), safflower seed oil (C), rapeseed oil (R), peanut oil (P) and soya bean oil (S), and five blended oils (B-1–B-5), representing each of the vegetable oils. Relative concentration of volatile compounds is indicated by color intensity, with brighter colors corresponding to higher concentrations.

In order to identify the volatile compounds that differed among different vegetable oil samples, the peaks obtained from HS-GC-IMS were selected for fingerprint comparison (Figure 3). GC-IMS revealed that the volatile compounds varied considerably among different vegetable oils, with 2-methyl-1-pentanol, E-3-Hexenol, p-methylacetophenone-M, 2-octanone, and methional mainly present in walnut oil, among which the relative percentage of alcohols was the highest at about 19.85%. Formaldehyde and 3-methylthiopropionaldehyde are mainly found in walnut oil, with the highest relative content of alcohols at about 19.85%. Studies have shown that walnut oil is rich in unsaturated fatty acids such as linoleic acid and oleic acid, which may be decomposed during oxidation to produce volatile compounds such as alcohols; at the same time, different varieties of walnut oil have different fatty acid compositions, and there may be differences in metabolic pathways, enzyme activities, etc., which may affect the synthesis of alcohols, and the relative content of alcohols produced in the process of oxidation may also be different [40]. The highest levels of esters and ketones were detected in safflower seed oil and rapeseed oil, including n-pentyl acetate, 2-furanmethanol acetate, 3-Methylbutyl butyrate, allyl (3-methylbutoxy) acetate, 5-methyl-3-heptanone and 3-methyl-2-pentanone. Linseed oil, sunflower oil and peanut oil showed high levels of 4-nonanone, 3-methyl-2-pentanone, heptanal, and methional.

The aroma substances of the blended oils contain a variety of components such as 4-ethylphenol, 2-Cyclohexen-1-one, furfural, Isobutyl 3-methylbutanoate, 4-hydroxy-5-ethyl-2-methyl-3(2H)-furanone, 3-octanol, 2-phenylethanal, valeric acid, 3-carene, 1-octen-3-ol, and linalool oxide. These aroma substances result from the combined contributions of the seven constituent vegetable oils. For instance, walnut oil imparts unique aroma components like 2-methyl-1-pentanol, while peanut oil and rapeseed oil contribute esters, ketones, and compounds such as acetophenone which are synergistically produced with linseed oil and sunflower oil. Sunflower oil further enriches the aroma profile through its esters and ketones. The blending process allows interactions between different oils, forming a complex and distinctive aroma combination.

#### 3.5.2. Multivariate Statistical Analysis

In this study, the orthogonal partial least squares discriminant analysis (OPLS-DA) model was used to identify the specific marker compounds for the differences in volatiles between vegetable oils and blended oils. The goodness-of-fit parameter (R^2^) and predictive ability parameter (Q^2^) of the model both approached 1.0, confirming its excellent stability and reliability for identifying and analyzing the differences in volatile components.

**Figure 4:**
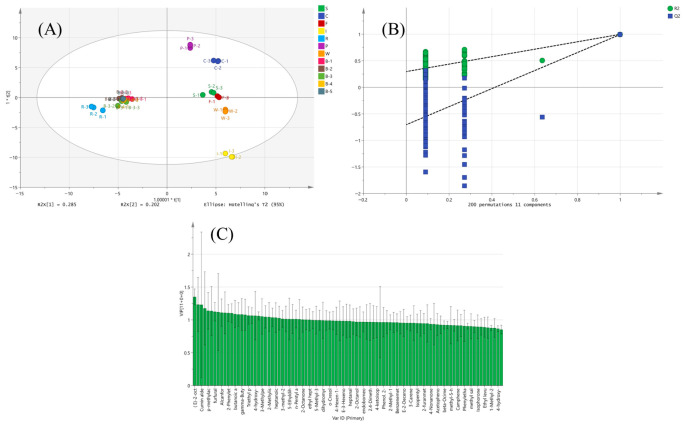
The OPLS-DA score scatter diagram (**A**) of the differences between vegetable oil samples; fig. (**B**) of the test results of 200 displacement tests; VIP Rating Chart (**C**). Walnut oil (W), linseed oil (I), sunflower oil (F), safflower seed oil (C), rapeseed oil (R), peanut oil (P) and soya bean oil (S), representing each of the vegetable oils.

As shown in the OPLS-DA score scatter plot (Figure 4A), distinct separation was observed among the different vegetable oil samples. Rapeseed oil is located in the third quadrant, peanut oil, soya bean oil and safflower seed oil are located in the second quadrant, sunflower oil is located in the junction of the second and fourth quadrants, and walnut oil and linseed oil are located in the fourth quadrant. Notably, the blended oil samples were found to cluster closely with rapeseed oil, which is consistent with the fingerprinting results (Figure 3) and likely due to the higher proportion of rapeseed oil in the blend formulation.

Subsequently, to identify the key aroma components contributing to the overall aroma profile of the vegetable oils, we screened for potential marker compounds based on the variable importance in projection (VIP) values derived from the OPLS-DA model (Figure 4C). Using the criteria of *p* < 0.05 and VIP > 1, a total of 19 different aroma substances were identified as key discriminants. These included five ketones, four esters, four alcohols, three aldehydes, one acid, and two other compounds, with higher VIP values indicating a greater contribution to the aroma characteristics of the samples [41].

#### 3.5.3. ROAV Analysis

The level of volatile compound content is not a sufficient absolute basis to determine the aroma characteristics of vegetable oils. The relative odor activity value (ROAV) explains how individual volatile compounds contribute to overall aroma based on their relative concentration [42]. Volatile compounds with ROAV ≥ 1 directly affect the sample’s overall aroma (key aroma-active compounds); those with 0.1 ≤ ROAV < 1 modify the aroma style (enhancing flavor characteristics); when ROAV < 0.1, they minimally affect the aroma style of the sample, even though there are synergistic or inhibitory effects among the aroma components, ROAV could be used to effectively evaluate their aroma contribution [43].

The odor threshold was determined for the differential volatile compounds in the samples and their ROAVs were calculated. This allowed the visualization of the contribution of different volatile compounds to aroma characteristics. The results showed (Table 4) that a total of ten differential volatile compounds had calculable ROAVs. The aroma of walnut oil was mainly influenced by the 4-hydroxy-5-ethyl-2-methyl-3(2H)-furanone, p-methylacetophenone-M, heptanal, 1-Heptanol, and presented coumarin, cherry, apigenin-like and other green herbaceous, fatty, sweet, woody, and peony aromas. Unlike walnut oil, the ROAVs of 1-Heptanol in the blended oils were all >3, and the ROAV of 1-Heptanol in walnut oil was 1.11, which indicated that the aromas of violet, herbaceous, sweet, woody, and peony brought by 1-Heptanol were enhanced by the blending. Moreover, the ROAV of 4-Ethylphenol in walnut oil was <1, and the ROAVs of 4-Ethylphenol in the five blended oils were all >1, which could be inferred from the effect of the addition of 1–5% of n-heptanol in the oils. It can be inferred that the addition of 1–5% linseed oil can bring phenolic, smoky and guaiacol flavors to the blended oils.

## 4. Conclusions

In the present study, the ideal ratio of the SFA:MUFA:PUFA ratio of 0.27:1:1 was achieved using a computer fitting technique. By adding high-oleic-acid vegetable oils (e.g., peanut oil, rapeseed oil), the MUFA content of the blended oils reached as high as 44.34% (2.23-fold higher than that of walnut oil), and the ω-6/ω-3 ratio was controlled in the range of 4–6:1. The acid value (0.53~0.80 mg/g) and peroxide value (2.89~8.91 meq/kg) of the blended oil were significantly lower than the national standard limit (acid value ≤ 3 mg/g, peroxide value ≤ 15.00 meq/kg). GC-IMS analysis showed that the blended oils developed a composite aroma distinguishing them from single oils, with p-methylacetophenone (almond aroma), 3-Methylbutyl butyrate (fruit aroma), and 4-Ethylphenol (smoky aroma) as the main aroma characteristics. In this study, multi-dimensional control strategies were used to achieve nutritional enhancement of mixed oils (optimization of dietary fatty acid composition and ω-6/ω-3 ratio), good physicochemical properties and flavor innovation of blended oils were achieved through a multi-dimensional control strategy, which opens up a new path for the development of functional fats and oils.

## Figures and Tables

**Table 1:** The optimal ratio of blend oil.

Group	Walnut Oil/%	Linseed Oil/%	Safflower Seed Oil/%	Sunflower Oil/%	Rapeseed Oil/%	Soya Oil/%	Peanut Oil/%
B-1	1	5	10	5	45	4	30
B-2	5	4	8	4	45	4	30
B-3	10	3	5	3	45	4	30
B-4	15	2	1	3	45	4	30
B-5	20	1	1	2	45	1	30

**Table 2:** Fatty acid composition, physicochemical properties and bioactive components of vegetable oil.

Sample	Walnut Oil	Linseed Oil	Sunflower Oil	Safflower Seed Oil	Rapeseed Oil	Soya Bean Oil	Peanut Oil
Fatty acid (%)							
C16:0	7.13 ± 0.41 ^c^	5.48 ± 0.01 ^d^	6.78 ± 0.06 ^c^	7.19 ± 0.06 ^c^	4.19 ± 0.00 ^e^	12.03 ± 0.11 ^a^	10.66 ± 0.06 ^b^
C18:0	2.50 ± 0.18 ^e^	5.21 ± 0.18 ^a^	3.55 ± 0.03 ^c^	3.03 ± 0.03 ^d^	2.17 ± 0.03 ^f^	3.89 ± 0.07 ^b^	5.42 ± 0.00 ^a^
C18:1n9c	13.65 ± 0.14 ^e^	19.41 ± 0.39 ^d^	19.06 ± 0.04 ^d^	11.45 ± 0.02 ^f^	61.05 ± 0.02 ^a^	21.26 ± 0.04 ^c^	41.34 ± 0.04 ^b^
C18:2n6c	63.70 ± 0.26 ^c^	14.92 ± 0.29 ^g^	69.30 ± 0.07 ^b^	76.92 ± 0.08 ^a^	20.12 ± 0.06 ^f^	53.59 ± 0.06 ^d^	36.79 ± 0.09 ^e^
C20:0	0.06 ± 0.00 ^f^	0.05 ± 0.01 ^f^	0.19 ± 0.00 ^e^	0.34 ± 0.00 ^c^	0.46 ± 0.00 ^b^	0.27 ± 0.01 ^d^	1.78 ± 0.00 ^a^
C20:1	ND	ND	0.10 ± 0.00 ^e^	0.13 ± 0.00 ^d^	0.97 ± 0.00 ^a^	0.22 ± 0.02 ^c^	0.58 ± 0.01 ^b^
C18:3n3	12.77 ± 0.08 ^b^	54.72 ± 0.01 ^a^	0.10 ± 0.01 ^e^	0.20 ± 0.00 ^e^	10.30 ± 0.01 ^c^	8.00 ± 0.09 ^d^	0.04 ± 0.00 ^e^
C22:0	ND	0.01 ± 0.00 ^e^	0.50 ± 0.00 ^b^	0.17 ± 0.00 ^d^	0.18 ± 0.00 ^d^	0.29 ± 0.00 ^c^	2.22 ± 0.00 ^a^
C24:0	ND	0.04 ± 0.00 ^e^	0.14 ± 0.00 ^b^	0.08 ± 0.00 ^d^	0.09 ± 0.01 ^c^	0.09 ± 0.00 ^c^	0.85 ± 0.00 ^a^
Mass parameter							
SFA	9.76	10.84	11.16	10.76	6.83	16.60	19.39
MUFA	13.72	19.48	19.14	11.55	61.17	21.32	41.41
PUFA	76.53	69.69	69.69	77.70	32.00	62.08	39.19
Omega-6	63.70	14.92	69.40	77.05	21.09	53.81	37.37
Omega-3	12.77	54.72	-	-	10.45	8.00	-
Omega-6/3	4.99	0.27	-	-	2.02	6.73	-
SFA:MUFA:PUFA	0.13:0.18:1	0.16:0.28:1	0.16:0.27:1	0.14:0.15:1	0.21:1.91:1	0.27:0.34:1	0.50:1.06:1
Physicochemical indicators							
IV/(g/100g)	128.24 ± 0.32 ^c^	147.13 ± 0.32 ^b^	107.72 ± 0.44 ^f^	185.96 ± 0.85 ^a^	109.06 ± 0.32 ^e^	114.28 ± 0.53 ^d^	87.63 ± 0.44 ^g^
SV/(mg/g)	182.32 ± 0.70 ^b^	185.36 ± 0.40 ^a^	141.89 ± 0.41 ^d^	143.05 ± 0.70 ^c^	118.98 ± 1.07 ^e^	103.78 ± 0.70 ^g^	116.64 ± 0.40 ^f^
AV/(mg NaOH/g)	0.11 ± 0.00 ^d^	0.35 ± 0.02 ^c^	0.10 ± 0.00 ^de^	1.50 ± 0.00 ^a^	0.93 ± 0.01 ^b^	0.08 ± 0.00 ^f^	0.10 ± 0.00 ^de^
POV/(meq/kg)	2.00 ± 0.00 ^g^	9.92 ± 0.14 ^b^	4.83 ± 0.14 ^d^	5.75 ± 0.00 ^c^	4.08 ± 0.14 ^f^	13.92 ± 0.14 ^a^	4.33 ± 0.14 ^e^
Minor nutrients (mg/100 g)							
VE	1.86 ± 0.03 ^f^	1.97 ± 0.17 ^f^	46.80 ± 3.60 ^b^	73.16 ± 0.61 ^a^	36.49 ± 3.21 ^c^	10.63 ± 2.79 ^e^	17.60 ± 1.77 ^d^
β-sitosterol	154.39 ± 11.00 ^d^	121.73 ± 9.88 ^e^	298.50 ± 8.84 ^b^	254.52 ± 2.10 ^c^	401.50 ± 10.70 ^a^	98.01 ± 22.63 ^e^	103.71 ± 3.81 ^e^
squalene	0.89 ± 0.03 ^d^	3.91 ± 0. 06 ^cd^	30.85 ± 1.34 ^b^	6.42 ± 2.39 ^c^	2.75 ± 0.07 ^cd^	31.31 ± 0.68 ^b^	37.21 ± 5.65 ^a^

Note: Results are presented as mean ± standard deviation (*n *= 3); Different lowercase letters (a–g) mean significant differences at the statistical level (*p* < 0.05); ND: Not detected.

**Table 3:** Fatty acid composition, physicochemical properties and bioactive components of blend oil.

Sample	Walnut Oil	B-1	B-2	B-3	B-4	B-5
C16:0	7.13 ± 0.41 ^a^	7.07 ± 0.00 ^a^	7.10 ± 0.14 ^a^	7.05 ± 0.05 ^a^	7.11 ± 0.01 ^a^	6.89 ± 0.02 ^a^
C18:0	2.50 ± 0.18 ^c^	3.55 ± 0.01 ^a^	3.49 ± 0.05 ^ab^	3.41 ± 0.02 ^ab^	3.37 ± 0.00 ^ab^	3.25 ± 0.01 ^b^
C18:1n9c	13.65 ± 0.14 ^d^	43.84 ± 0.10 ^a^	43.91 ± 0.12 ^a^	43.63 ± 0.03 ^b^	43.59 ± 0.00 ^b^	43.30 ± 0.02 ^c^
C18:2n6c	63.70 ± 0.26 ^a^	35.24 ± 0.02 ^cd^	35.12 ± 0.01 ^d^	35.51 ± 0.02 ^c^	35.50 ± 0.00 ^c^	36.32 ± 0.01 ^b^
C20:0	0.06 ± 0.00 ^d^	0.81 ± 0.00 ^a^	0.81 ± 0.01 ^a^	0.79 ± 0.02 ^ab^	0.77 ± 0.00 ^bc^	0.75 ± 0.00 ^c^
C20:1	ND	0.51 ± 0.01 ^a^	0.55 ± 0.05 ^a^	0.56 ± 0.02 ^a^	0.57 ± 0.07 ^a^	0.49 ± 0.06 ^a^
C18:3n3	12.77 ± 0.08 ^a^	7.78 ± 0.11 ^b^	7.83 ± 0.03 ^b^	7.89 ± 0.07 ^b^	7.96 ± 0.05 ^b^	7.89 ± 0.05 ^b^
C22:0	ND	0.85 ± 0.00 ^a^	0.85 ± 0.01 ^a^	0.83 ± 0.01 ^b^	0.81 ± 0.00 ^c^	0.78 ± 0.01 ^d^
C24:0	ND	0.34 ± 0.00 ^a^	0.34 ± 0.00 ^a^	0.33 ± 0.01 ^b^	0.32 ± 0.01 ^b^	0.32 ± 0.00 ^b^
Mass parameter						
SFA	9.76	11.81	11.78	11.62	11.61	11.24
MUFA	13.72	43.84	43.91	43.63	43.59	43.30
PUFA	76.53	44.34	44.31	44.75	44.80	45.45
Omega-6	63.70	35.75	35.67	36.07	36.07	36.81
Omega-3	12.77	7.78	7.83	7.89	7.96	7.89
Omega-6/3	4.99	4.60	4.56	4.57	4.53	4.67
SFA:MUFA:PUFA	0.13:0.18:1	0.266:0.988:1	0.265:0.99:1	0.259:0.974:1	0.259:0.972:1	0.247:0.952:1
Physicochemical indicators						
IV/(g/100g)	128.24 ± 0.32 ^a^	110.69 ± 0.44 ^b^	108.15 ± 0.63 ^c^	94.89 ± 0.12 ^e^	93.84 ± 0.53 ^f^	101.80 ± 0.53 ^d^
SV/(mg/g)	182.32 ± 0.70 ^e^	222.20 ± 1.26 ^b^	198.45 ± 0.70 ^d^	247.77 ± 0.41 ^a^	207.34 ± 0.41 ^c^	198.45 ± 0.70 ^d^
AV/(mg NaOH/g)	0.11 ± 0.00 ^e^	0.80 ± 0.00 ^a^	0.78 ± 0.00 ^b^	0.78 ± 0.00 ^b^	0.56 ± 0.00 ^c^	0.53 ± 0.00 ^d^
POV/(meq/kg)	2.00 ± 0.00 ^e^	2.89 ± 0.12 ^d^	8.91 ± 0.14 ^a^	4.91 ± 0.14 ^c^	7.58 ± 0.25 ^b^	4.91 ± 0.14 ^c^
Minor nutrients (mg/100 g)						
VE	1.86 ± 0.03 ^a^	3.51 ± 0.53 ^a^	3.03 ± 1.02 ^a^	2.95 ± 1.04 ^a^	3.19 ± 0.01 ^a^	3.93 ± 0.01 ^a^
β-sitosterol	154.39 ± 11.00 ^a^	96.70 ± 16.07 ^b^	77.40 ± 10.63 ^b^	26.13 ± 0.88 ^c^	50.11 ± 4.98 ^c^	34.10 ± 5.87 ^c^
squalene	0.89 ± 0.03 ^b^	1.95 ± 0.77 ^a^	1.57 ± 0.04 ^ab^	1.94 ± 0.24 ^a^	2.07 ± 0.22 ^a^	2.28 ± 0.06 ^a^

Note: Results are presented as mean ± standard deviation (*n *= 3); Different lowercase letters (a–f) mean significant differences at the statistical level (*p* < 0.05); ND: Not detected.

**Table 4:** ROAVs of different volatile compounds in different vegetable oils.

VOCs	Threshold Value (Tx, mg/kg)	Odor Description	ROAV											
			Walnut Oil	Linseed Oil	Sunflower Oil	Safflower Seed Oil	Rapeseed Oil	Soya Bean Oil	Peanut Oil	B-1	B-2	B-3	B-4	B-5
Isopentyl propanoate	0.7	bananas, pineapples, mature tropical fruits	0.31	1.40	0.18	0.15	0.43	0.65	0.11	0.51	0.47	0.47	0.41	0.36
butyl propanoate	/	fruity, sweet, earthy fragrance rose flavor	/	/	/	/	/	/	/	/	/	/	/	/
dihydromyrcenyl acetate	/	citrus, bergamot orange, lavender	/	/	/	/	/	/	/	/	/	/	/	/
3-Methylbutyl butyrate	/	apricot, pear, banana	/	/	/	/	/	/	/	/	/	/	/	/
4-hydroxy-5-ethyl-2-methyl-3(2H)-furanone	0.0025	bread aroma, caramel aroma, sweet aroma, fruit aroma	100.00	95.51	100.00	50.78	50.32	80.33	33.93	50.76	48.35	66.55	54.35	48.87
3-methyl-2-pentanone	/	/	/	/	/	/	/	/	/	/	/	/	/	/
p-methylacetophenone (M)	0.0156	sweet, creamy, mimosa-like, coumarin, cherry and apigenin-like flavors	84.95	100.00	55.67	100.00	100.00	100.00	100.00	100.00	100.00	100.00	100.00	100.00
4-Nonanone	/	/	/	/	/	/	/	/	/	/	/	/	/	/
2-Cyclohexen-1-one	/	barbecue, mint, wood	/	/	/	/	/	/	/	/	/	/	/	/
heptanal	0.031	fresh aldehydes, fat, green herbs, alfalfa parsley flavor	3.89	31.01	6.22	3.47	28.77	25.42	11.90	16.74	17.06	15.71	16.85	16.21
Piperonal propylene glycol acetal	/	/	/	/	/	/	/	/	/	/	/	/	/	/
furfural	8	sweet, almond flavor, toast flavor, wood flavor, caramel flavor, with burnt astringency.	0.01	0.03	0.01	0.01	0.01	0.01	0.01	0.07	0.06	0.08	0.06	0.07
Benzenemethanol	5.5	aroma of flowers, roses, almonds, balsam, bitterness	0.02	0.07	0.03	0.04	0.08	0.13	0.02	0.06	0.06	0.06	0.06	0.06
1-Heptanol	0.2	violet, herbs, sweet, wood, peony	1.11	3.59	1.03	0.84	4.80	5.51	0.51	3.15	3.15	3.48	3.32	3.25
E-3-Hexenol	1.5	green, leathery, floral, oily, earthy.	0.69	1.04	0.22	0.28	0.69	1.70	0.08	0.62	0.62	0.69	0.67	0.63
2-Methyl-1-pentanol	/	/	/	/	/	/	/	/	/	/	/	/	/	/
butanoic acid	2.682	acidic sour, cheese, dairy, creamy and fruity flavors	0.21	0.22	0.05	0.08	0.15	0.26	0.07	0.10	0.10	0.10	0.10	0.10
4-Ethylphenol	0.13	phenolic, smoked, guaiacol	0.70	1.23	0.45	0.49	0.90	0.65	0.73	1.08	1.48	1.60	1.45	1.34
Camphene	/	camphor, cool, mint, citrus and green spicy nuances.	/	/	/	/	/	/	/	/	/	/	/	/

Note: Odor descriptions were cited from http://www.thegoodscentscompany.com (accessed on 8 December 2025) and https://mffi.sjtu.edu.cn/database (accessed on 8 December 2025).

## Data Availability

The original contributions presented in this study are included in the article/Supplementary Material. Further inquiries can be directed to the corresponding authors.

## Supplementary Materials

The following supporting information can be downloaded at: https://www.mdpi.com/article/10.3390/foods15111840/s1. Figure S1: Vitamin E standard curve (**A**), β-sitosterol standard curve (**B**), squalene standard curve (**C**). Figure S2: GC-IMS two-dimensional chromatograms (**A**) of volatile components in vegetable oil samples; GC-IMS two-dimensional spectrum of volatile components in blend oil samples (**B**). Table S1: Electronic nose sensor response value. Table S2: Relative content of volatile components in vegetable oil and blend oil.

## Abbreviations

SFA, saturated fatty acid; MUFA, monounsaturated fatty acid; PUFA, polyunsaturated fatty acid; ω-6, omega-6 fatty acids; ω-3, omega-3 fatty acids; FFA, free fatty acid; IV, Iodine value; POV, peroxide value; AV, acid value (AV);W, walnut oil; I, linseed oil; F, sunflower seed oil; C, safflower seed oil; S, soya oil; P, peanut oil; R, rapeseed oil; VOCs, volatile organics; WHO, world health organization; HPLC, high performance liquid chromatography; HS-GC-IMS, headspace-gas chromatography-ion mobility spectrometry; PCA, principal component analysis; OPLS-DA, orthogonal partial least squares discriminant analysis; OAV, odor activity value; ROAV, relative odor activity value.
