# Supplementary material for: Physicochemical Properties, Bioactive Components and Volatile Compounds of Dietary Fatty Acid Balanced Blend Oil
Source: Foods. 2026 May 22;15(11):1840. doi: 10.3390/foods15111840 (PMC13257371; doi:10.3390/foods15111840)
Supplement: Supplementary file 1 [file foods-15-01840-s001.zip › Table S1.pdf]

Table S1 Electronic nose sensor response value

	Walnut oil	Linseed oil	Sunflower oil	Safflower seed oil	Rapeseed oil	Soya bean oill	Peanut oil	B-1	B-2	B-3	B-4	B-5
W1C	0.62±0.01	0.59±0.01	0.35±0.03	0.64±0.02	0.49±0.02	0.52±0.01	0.53±0.02	0.54±0.01	0.56±0.02	0.59±0.02	0.63±0.00	0.59±0.06
W5S	2.72±0.14	2.79±0.09	4.74±0.53	2.39±0.02	3.35±0.07	3.04±0.08	3.10±0.11	3.07±0.04	2.89±0.12	2.92±0.14	2.67±0.05	2.79±0.22
W3C	0.91±0.00	0.89±0.00	0.74±0.03	0.90±0.01	0.84±0.02	0.86±0.01	0.86±0.01	0.87±0.00	0.88±0.01	0.90±0.01	0.91±0.00	0.90±0.03
W6S	1.10±0.03	1.04±0.01	1.19±0.04	1.19±0.05	1.18±0.02	1.10±0.07	1.81±1.14	1.10±0.05	1.10±0.03	1.04±0.01	1.05±0.03	1.03±0.01
W5C	0.97±0.00	0.96±0.00	0.85±0.03	0.96±0.01	0.93±0.01	0.94±0.00	0.94±0.01	0.95±0.00	0.96±0.01	0.96±0.00	0.97±0.00	0.97±0.01
W1S	2.94±0.04	3.14±0.09	7.48±0.82	2.92±0.13	4.42±0.37	3.63±0.25	4.20±0.61	3.59±0.16	3.27±0.32	3.09±0.15	2.74±0.07	2.98±0.52
W1W	3.96±0.18	4.82±0.64	7.70±0.97	3.04±0.02	5.17±0.17	4.45±0.30	4.30±0.27	4.81±0.12	4.27±0.23	4.56±0.27	3.87±0.18	4.01±0.44
W2S	2.51±0.04	2.70±0.12	6.57±1.08	2.47±0.1	3.63±0.25	3.19±0.04	3.47±0.40	3.01±0.09	2.81±0.25	2.62±0.12	2.36±0.05	2.63±0.51
W2W	3.33±0.18	3.66±0.30	7.16±1.3	2.34±0.02	4.03±0.07	3.25±0.20	2.92±0.18	3.30±0.07	2.94±0.12	3.18±0.18	2.74±0.10	2.76±0.23
W3S	1.06±0.01	1.04±0.02	1.09±0.04	1.10±0.02	1.09±0.03	1.08±0.03	1.09±0.02	1.05±0.01	1.07±0.04	1.05±0.01	1.04±0.01	1.03±0.02

*Results are presented as mean ± standard deviation (n=3).
