# Supplementary material for: Physicochemical Properties, Bioactive Components and Volatile Compounds of Dietary Fatty Acid Balanced Blend Oil
Source: Foods. 2026 May 22;15(11):1840. doi: 10.3390/foods15111840 (PMC13257371; doi:10.3390/foods15111840)
Supplement: Supplementary file 1 [file foods-15-01840-s001.zip › Table S2.pdf]

Table S2 Relative content of volatile components in vegetable oil and blend oil

VOCs	Comparative content /%											
	Walnut oil	Linseed oil	Sunflower oil	Safflower seed oil	Rapeseed oil	Soya bean oill	Peanut oil	B-1	B-2	B-3	B-4	B-5
**Ester**												
Ethyl benzoate	0.605±0.009	0.464±0.004	0.91±0.037	0.687±0.014	0.997±0.042	0.665±0.185	0.527±0.009	0.745±0.014	0.712±0.009	0.705±0.012	0.719±0.005	0.723±0.003
Benzyl formate	0.526±0.011	0.627±0.028	0.556±0.004	0.513±0.012	0.527±0.012	0.777±0.017	0.525±0.008	0.487±0.014	0.481±0.004	0.497±0.018	0.49±0.003	0.484±0.005
Ethyl levulinate	0.478±0.007	0.43±0.007	0.544±0.018	0.635±0.012	0.931±0.037	0.707±0.097	0.633±0.003	0.746±0.01	0.762±0.009	0.75±0.013	0.744±0.006	0.753±0.007
Isopentyl propanoate	1.001±0.012	2.613±0.013	0.78±0.009	0.53±0.011	1.018±0.036	1.449±0.017	0.443±0.01	1.341±0.012	1.24±0.011	1.186±0.048	1.054±0.003	0.932±0.005
butyl propanoate	2.64±0.021	3.439±0.006	2.068±0.033	2.189±0.007	2.316±0.026	3.895±0.019	0.882±0.008	2.833±0.018	2.76±0.009	2.749±0.06	2.511±0.007	2.496±0.005
n-Pentyl acetate	2.751±0.017	3.837±0.017	5.19±0.027	8.199±0.047	2.49±0.01	3.599±0.026	3.56±0.021	2.685±0.016	2.503±0.019	1.916±0.019	1.715±0.011	1.802±0.02
gamma-Butyrolactone	1.192±0.02	0.44±0.004	0.978±0.092	1.864±0.059	0.691±0.015	1.044±0.024	2.151±0.064	0.857±0.025	0.933±0.021	0.777±0.016	0.755±0.012	0.782±0.021
Ethyl 3-hydroxybutanoate	1.195±0.041	1.148±0.012	1.375±0.095	1.406±0.041	1.855±0.060	2.197±0.022	2.994±0.034	1.802±0.026	1.900±0.021	1.725±0.03	1.83±0.033	1.804±0.021
2-furanmethanol acetate	1.884±0.084	0.775±0.018	2.599±0.081	4.025±0.036	2.106±0.053	0.677±0.054	4.728±0.135	1.873±0.042	1.997±0.013	1.919±0.044	1.971±0.008	2.109±0.009

Allyl (3-methylbutoxy)acetate	0.274±0.002	0.314±0.003	0.412±0.019	0.327±0.006	0.908±0.015	0.295±0.008	0.419±0.005	0.594±0.017	0.572±0.011	0.578±0.024	0.549±0.008	0.569±0.005
dihydromyrcenyl acetate	0.237±0.002	0.423±0.006	2.591±0.023	0.254±0.013	0.201±0.004	0.315±0.010	0.545±0.010	0.497±0.007	0.43±0.004	0.397±0.020	0.389±0.003	0.334±0.010
ethyl heptanoate	0.308±0.014	0.546±0.006	0.234±0.002	0.231±0.005	0.331±0.017	0.229±0.005	0.266±0.010	0.26±0.007	0.249±0.002	0.270±0.010	0.259±0.003	0.245±0.009
methyl salicylate	0.258±0.004	0.277±0.009	0.228±0.005	0.237±0.006	0.84±0.003	0.273±0.007	0.305±0.018	0.503±0.006	0.498±0.005	0.518±0.029	0.502±0.004	0.512±0.012
sotolon	0.206±0.002	0.195±0.002	0.19±0.011	0.269±0.005	0.649±0.046	0.236±0.008	0.257±0.007	0.522±0.024	0.486±0.015	0.502±0.039	0.492±0.01	0.507±0.008
3-Methylbutyl butyrate	0.426±0.015	0.414±0.004	0.655±0.021	0.272±0.014	3.654±0.028	0.447±0.005	0.244±0.012	2.82±0.078	2.895±0.005	2.878±0.044	2.958±0.006	2.928±0.007
5-Ethyldihydro-2(3H)-furanone	0.639±0.006	0.753±0.014	0.740±0.020	0.921±0.072	1.271±0.042	0.826±0.049	0.915±0.013	0.929±0.018	0.912±0.018	0.912±0.028	0.889±0.005	0.909±0.007
Isobutyl 3-methylbutanoate	0.327±0.014	0.529±0.009	0.437±0.015	0.342±0.006	0.811±0.019	0.323±0.008	0.329±0.037	0.832±0.087	0.764±0.007	0.801±0.041	0.799±0.004	0.823±0.012
beta-methyl-gamma-octalactone	0.438±0.016	0.266±0.001	0.284±0.008	0.28±0.023	0.205±0.002	0.2±0	0.32±0.011	0.29±0.009	0.367±0.028	0.394±0.033	0.392±0.016	0.399±0.002
Triethyl phosphate	0.479±0.011	0.412±0.006	0.353±0.006	0.389±0.031	0.353±0.010	0.294±0.008	0.312±0.009	0.443±0.050	0.571±0.034	0.598±0.038	0.575±0.018	0.546±0.020
**Ketone**												
Isophorone oxide	0.725±0.021	0.88±0.014	1.063±0.049	1.118±0.011	1.739±0.032	0.800±0.003	1.069±0.023	1.433±0.015	1.376±0.021	1.361±0.028	1.373±0.008	1.401±0.019

4-ketoisophorone	1.04±0.074	1.841±0.031	1.769±0.047	2.49±0.013	1.769±0.011	2.431±0.03	2.84±0.039	1.871±0.012	1.829±0.017	1.748±0.016	1.753±0.019	1.737±0.011
4-hydroxy-5-ethyl-2-methyl-3(2H)-furanone	1.166±0.041	0.637±0.050	1.563±0.064	0.625±0.029	0.423±0.019	0.637±0.034	0.488±0.012	0.48±0.072	0.453±0.001	0.600±0.116	0.503±0.004	0.458±0.015
Acetophenone	6.179±0.029	4.164±0.075	5.428±0.072	7.686±0.035	5.242±0.087	4.950±0.035	8.969±0.180	5.899±0.032	5.848±0.027	5.628±0.099	5.778±0.027	5.851±0.017
methyl-5-hepten-2-one	1.151±0.024	0.582±0.008	1.06±0.022	1.779±0.034	0.886±0.007	0.975±0.006	2.757±0.054	1.116±0.012	1.176±0.020	1.106±0.035	1.159±0.005	1.161±0.005
5-Methyl-3-heptanone	1.086±0.015	0.944±0.011	1.098±0.070	2.131±0.013	0.882±0.033	1.068±0.025	1.284±0.046	0.99±0.026	0.886±0.016	0.878±0.023	0.888±0.010	0.917±0.011
3-methyl-2-pentanone	1.333±0.034	4.045±0.01	1.527±0.018	1.888±0.035	2.667±0.027	1.590±0.029	2.028±0.021	1.861±0.005	1.786±0.007	1.596±0.018	1.595±0.006	1.48±0.007
Cyclohexanone	1.133±0.108	0.67±0.009	1.188±0.323	1.003±0.049	1.096±0.010	0.74±0.016	1.718±0.037	0.942±0.008	0.976±0.026	0.947±0.015	1.048±0.005	1.040±0.010
p-methylacetophenone(M)	0.293±0.006	0.799±0.016	2.212±0.019	0.316±0.027	0.256±0.001	0.268±0.014	0.308±0.020	1.021±0.005	0.829±0.022	0.736±0.018	0.74±0.007	0.591±0.010
methylacetophenone(D)	0.207±0.003	0.197±0.000	1.363±0.025	0.232±0.008	0.174±0.003	0.224±0.005	0.272±0.006	0.212±0.003	0.21±0.011	0.197±0.001	0.201±0.004	0.194±0.002
3-Ethyl-2-hydroxycyclopent-2-en-1-one	0.246±0.011	0.483±0.015	0.574±0.007	0.373±0.009	0.670±0.026	0.339±0.056	0.312±0.002	0.372±0.006	0.353±0.003	0.354±0.014	0.335±0.005	0.34±0.015
4-Nonanone	1.263±0.029	2.428±0.194	2.550±0.037	0.598±0.012	0.935±0.028	0.510±0.009	0.356±0.023	0.813±0.035	0.729±0.013	0.738±0.044	0.742±0.007	0.755±0.012

1-Methyl-2,3-Cyclohexanedione	0.449±0.014	0.875±0.012	0.356±0.036	0.315±0.017	0.889±0.020	0.326±0.005	0.497±0.040	0.697±0.001	0.697±0.013	0.695±0.006	0.681±0.007	0.669±0.009
Isophorone	0.725±0.021	0.88±0.014	1.063±0.049	1.118±0.011	1.739±0.032	0.800±0.003	1.069±0.023	1.433±0.015	1.376±0.021	1.361±0.028	1.373±0.008	1.401±0.019
4-hydroxy-2,5-dimethyl-3(2 H)-furanone	0.407±0.019	0.364±0.002	0.319±0.005	0.439±0.009	0.894±0.041	0.421±0.019	0.457±0.006	0.82±0.014	0.849±0.011	0.837±0.010	0.871±0.005	0.868±0.005
2-Octanone	0.960±0.115	0.562±0.015	0.684±0.056	0.483±0.007	0.559±0.003	0.422±0.007	0.390±0.070	0.533±0.090	0.415±0.011	0.488±0.020	0.498±0.008	0.531±0.003
Alcanfor	0.299±0.002	0.479±0.003	0.387±0.002	0.439±0.036	0.373±0.005	0.368±0.014	0.550±0.005	0.547±0.062	0.694±0.047	0.694±0.025	0.640±0.019	0.598±0.015
2-Cyclohexen-1-one	0.174±0.005	1.128±0.067	0.230±0.007	0.180±0.004	0.782±0.014	0.488±0.014	0.298±0.006	2.452±0.018	2.390±0.014	2.790±0.074	2.277±0.004	2.393±0.002
**Aldehydes**												
Cumin aldehyde	0.221±0.005	0.282±0.006	0.203±0.003	0.242±0.004	0.346±0.004	0.352±0.123	0.2630±0.006	0.265±0.003	0.266±0.004	0.270±0.009	0.268±0.006	0.265±0.007
(E)-2-Nonenal	0.355±0.01	0.437±0.055	0.435±0.005	0.677±0.013	0.323±0.007	0.518±0.004	0.428±0.004	0.358±0.01	0.333±0.003	0.322±0.002	0.323±0.002	0.326±0.008
Methyl-5-furfural	0.428±0.016	0.390±0.002	0.585±0.020	0.991±0.006	0.804±0.051	1.559±0.032	0.883±0.017	0.665±0.025	0.711±0.009	0.700±0.010	0.745±0.003	0.750±0.007
2-Phenylethanal	0.929±0.028	0.937±0.017	0.844±0.023	1.156±0.019	1.060±0.009	1.436±0.027	0.627±0.062	1.090±0.024	1.183±0.023	1.125±0.024	1.162±0.003	1.172±0.004

heptanal	0.562±0.015	2.566±0.006	1.205±0.032	0.530±0.089	2.998±0.010	2.501±0.008	2.120±0.041	1.962±0.005	1.982±0.008	1.757±0.01	1.934±0.016	1.885±0.003
(E)-2-octenal	0.53±0.006	0.591±0.033	0.486±0.009	0.837±0.010	0.352±0.002	0.552±0.015	0.505±0.005	0.459±0.035	0.589±0.045	0.623±0.011	0.608±0.011	0.573±0.017
3-methyl-2-butenal	1.319±0.003	1.458±0.021	1.321±0.005	2.809±0.087	1.308±0.011	1.198±0.021	3.983±0.034	1.967±0.014	2.066±0.017	2.07±0.113	2.115±0.016	2.064±0.009
2,4-Dimethylbenzaldehyde	0.208±0.001	0.200±0.005	0.789±0.019	0.229±0.003	0.176±0.003	0.231±0.007	0.275±0.004	0.203±0.001	0.199±0.006	0.200±0.004	0.199±0.003	0.195±0.005
2,4-heptadienal (E,E)	0.176±0.006	0.232±0.007	0.155±0.001	0.199±0.001	0.652±0.013	0.210±0.010	0.245±0.003	0.735±0.011	0.692±0.013	0.689±0.020	0.684±0.001	0.708±0.011
p-Anisaldehyde	0.672±0.021	0.236±0.009	0.280±0.009	0.268±0.007	0.183±0.005	0.226±0.003	0.297±0.012	0.213±0.012	0.213±0.007	0.222±0.01	0.230±0.007	0.246±0.008
Piperonal propylene glycol aceta	6.889±0.017	3.181±0.098	5.996±0.021	5.510±0.065	3.841±0.015	9.346±0.040	5.891±0.053	4.537±0.068	4.422±0.029	5.077±0.294	5.234±0.016	5.137±0.016
furfural	0.415±0.009	0.712±0.011	0.191±0.007	0.182±0.006	0.205±0.017	0.163±0.008	0.232±0.009	2.022±0.011	1.91±0.023	2.27±0.073	1.892±0.022	2.091±0.009

Methional	5.482±0.080	1.649±0.005	4.799±0.023	2.486±0.013	4.530±0.038	1.258±0.021	2.624±0.027	4.151±0.005	4.349±0.020	4.321±0.103	4.572±0.010	4.672±0.023
**Alcohols**												
Maltol	0.261±0.01	0.367±0.007	0.265±0.009	0.507±0.141	0.258±0.012	0.814±0.173	0.375±0.003	0.255±0.007	0.248±0.003	0.256±0.012	0.252±0.005	0.244±0.001
2-Methylisoborneol	1.529±0.013	0.647±0.005	0.794±0.024	0.779±0.014	0.439±0.011	0.372±0.006	0.785±0.011	0.518±0.063	0.509±0.008	0.548±0.027	0.616±0.013	0.702±0.004
2-Phenethyl alcohol	0.361±0.038	1.871±0.005	1.508±0.032	2.193±0.005	1.354±0.066	1.705±0.095	1.642±0.035	1.286±0.036	1.152±0.038	1.059±0.04	0.993±0.015	0.968±0.01
Linalool oxide	1.758±0.011	1.381±0.004	1.598±0.059	2.223±0.009	1.620±0.050	2.187±0.020	2.978±0.077	1.765±0.004	1.798±0.010	1.733±0.070	1.779±0.009	1.783±0.012
Benzenemethanol	0.515±0.025	1.089±0.015	1.086±0.021	0.979±0.119	1.482±0.008	2.289±0.024	0.653±0.040	1.266±0.028	1.276±0.004	1.194±0.015	1.234±0.004	1.225±0.018
3-Octanol	1.016±0.024	0.994±0.004	1.400±0.033	1.388±0.004	1.967±0.027	1.339±0.009	1.134±0.013	1.630±0.024	1.660±0.011	1.661±0.004	1.690±0.014	1.701±0.004
1-Heptanol	1.036±0.013	1.918±0.134	1.287±0.019	0.824±0.013	3.224±0.004	3.496±0.021	0.580±0.023	2.382±0.015	2.359±0.017	2.509±0.094	2.459±0.011	2.440±0.010
E-3-Hexenol	4.806±0.023	4.180±0.006	2.084±0.029	2.075±0.028	3.470±0.053	8.093±0.059	0.669±0.035	3.493±0.013	3.467±0.033	3.724±0.061	3.727±0.024	3.524±0.003
1-Octen-3-ol	1.326±0.055	0.834±0.006	1.157±0.024	1.448±0.026	1.207±0.034	1.409±0.019	2.181±0.051	1.222±0.007	1.273±0.011	1.232±0.042	1.249±0.006	1.273±0.002

4-Hexen-1-ol	1.089±0.011	0.696±0.021	0.805±0.01	1.090±0.037	0.693±0.003	1.598±0.085	0.629±0.023	0.680±0.019	0.639±0.002	0.684±0.037	0.692±0.017	0.681±0.004
1-nonanol	0.245±0.011	0.265±0.011	0.617±0.031	0.229±0.004	0.259±0.012	0.215±0.008	0.259±0.001	0.255±0.016	0.230±0.003	0.235±0.005	0.233±0.001	0.235±0.002
2-Methyl-1-pentanol	4.337±0.01	2.561±0.07	2.814±0.006	0.388±0.016	2.493±0.044	0.364±0.008	0.896±0.009	1.788±0.008	1.883±0.010	1.912±0.041	2.184±0.011	2.254±0.008
3-endoborneol	0.418±0.006	0.527±0.013	0.433±0.02	0.309±0.011	0.260±0.005	0.252±0.01	0.342±0.012	0.302±0.006	0.312±0.013	0.318±0.008	0.309±0.006	0.31±0.003
2-Octanol	1.149±0.003	0.355±0.006	0.489±0.008	0.267±0.01	0.487±0.013	0.252±0.002	0.274±0.002	0.482±0.008	0.481±0.011	0.524±0.008	0.559±0.011	0.572±0.007
**Acid**												
E-2-Decenoic acid	0.389±0.004	0.343±0.008	0.361±0.014	0.413±0.018	0.616±0.01	0.419±0.023	0.47±0.025	0.454±0.01	0.47±0.009	0.458±0.022	0.455±0.009	0.451±0.003
heptanoic acid	0.802±0.003	0.727±0.035	0.812±0.048	1.151±0.026	0.598±0.037	0.731±0.013	0.813±0.055	0.625±0.015	0.571±0.015	0.558±0.007	0.551±0.005	0.557±0.012
Hexanoic acid	3.15±0.021	1.74±0.004	1.904±0.018	1.546±0.164	1.992±0.024	0.917±0.032	1.031±0.017	2.004±0.007	2.031±0.003	2.074±0.013	2.121±0.008	2.161±0.013
Pentanoic acid	0.886±0.019	0.946±0.036	0.882±0.043	0.845±0.023	0.692±0.007	1.231±0.002	0.828±0.006	0.710±0.011	0.760±0.007	0.831±0.034	0.843±0.007	0.829±0.003
butanoic acid	2.682±0.011	1.567±0.092	0.921±0.008	1.025±0.026	1.346±0.057	2.226±0.01	1.127±0.06	1.018±0.008	0.995±0.023	0.978±0.027	1.008±0.012	0.974±0.004

3-methylbutyric acid	1.710±0.016	1.529±0.085	1.934±0.058	1.207±0.024	1.380±0.011	0.724±0.009	0.904±0.019	1.124±0.016	1.069±0.014	1.107±0.046	1.130±0.020	1.138±0.011
3-Methylpentanoic acid	1.024±0.007	0.948±0.024	1.056±0.039	1.603±0.025	0.784±0.039	0.865±0.003	1.153±0.023	0.835±0.026	0.765±0.004	0.788±0.026	0.794±0.016	0.815±0.009
**Phenols**												
o-Cresol	1.465±0.015	1.698±0.011	0.565±0.006	0.664±0.009	0.589±0.036	0.681±0.008	0.640±0.036	0.927±0.005	0.905±0.023	0.856±0.025	0.830±0.004	0.811±0.002
4-ethyl-2-methoxyphenol	0.315±0.017	0.234±0.028	0.415±0.027	0.265±0.003	0.192±0.007	0.205±0.007	0.355±0.025	0.230±0.027	0.218±0.007	0.216±0.005	0.222±0.003	0.224±0.005
p-Methyl guaiacol	0.282±0.008	0.745±0.019	0.277±0.037	0.264±0.018	0.305±0.008	0.259±0.007	0.345±0.03	0.304±0.026	0.369±0.012	0.392±0.029	0.364±0.004	0.350±0.001
4-Ethylphenol	0.424±0.011	0.426±0.000	0.362±0.032	0.316±0.016	0.391±0.007	0.266±0.021	0.545±0.045	0.533±0.09	0.723±0.028	0.748±0.047	0.699±0.028	0.655±0.01
**Others**												
beta-Ocimene	0.703±0.007	0.927±0.012	0.87±0.012	1.214±0.062	0.921±0.009	1.671±0.01	1.469±0.015	1.058±0.012	1.079±0.013	1.037±0.042	1.050±0.006	1.035±0.01
Myrcene	0.816±0.025	0.821±0.039	0.595±0.043	0.656±0.037	0.644±0.028	1.246±0.002	1.536±0.173	0.631±0.027	0.600±0.004	0.666±0.098	0.705±0.024	0.714±0.035
isopropylbenzene	0.811±0.011	0.409±0.004	0.517±0.011	0.996±0.038	0.343±0.014	0.497±0.013	0.497±0.018	0.422±0.012	0.475±0.03	0.496±0.019	0.483±0.008	0.462±0.007
3-Carene	0.877±0.048	0.641±0.024	0.818±0.048	1.037±0.013	0.847±0.01	0.742±0.008	1.118±0.025	0.902±0.028	0.938±0.008	0.871±0.025	0.888±0.015	0.890±0.014

Camphene	3.229±0.024	6.535±0.008	0.894±0.005	1.089±0.021	1.524±0.04	0.823±0.063	0.813±0.082	1.452±0.015	1.453±0.012	1.407±0.059	1.431±0.007	1.458±0.018
2-Butanoyl furan	0.193±0.002	0.183±0.002	0.170±0.005	0.218±0.004	1.038±0.002	0.218±0.007	0.26±0.006	0.445±0.009	0.422±0.009	0.447±0.030	0.453±0.003	0.458±0.007
cis-Rose oxide	0.280±0.006	0.375±0.007	0.229±0.011	0.283±0.01	0.292±0.007	0.232±0.008	0.267±0.009	0.359±0.025	0.469±0.032	0.477±0.023	0.452±0.016	0.44±0.014
1,2,4,5-tetramethylbenzene	1.462±0.021	1.841±0.009	1.058±0.025	1.135±0.014	0.575±0.019	0.908±0.02	0.742±0.004	0.762±0.017	0.724±0.008	0.725±0.016	0.721±0.002	0.735±0.005
1,2,3-trimethylbenzene	1.462±0.021	1.841±0.009	1.058±0.025	1.135±0.014	0.575±0.019	0.908±0.02	0.742±0.004	0.762±0.017	0.724±0.008	0.725±0.016	0.721±0.002	0.735±0.005
p-Cymene	0.599±0.011	0.565±0.004	0.501±0.021	0.597±0.034	0.378±0.004	0.641±0.005	0.358±0.008	0.418±0.012	0.403±0.015	0.420±0.007	0.406±0.006	0.396±0.006

*Results are presented as mean ± standard deviation (n=3).
