# Supplementary figures and images for: Physicochemical Properties, Bioactive Components and Volatile Compounds of Dietary Fatty Acid Balanced Blend Oil
Source: Foods. 2026 May 22;15(11):1840. doi: 10.3390/foods15111840 (PMC13257371; doi:10.3390/foods15111840)

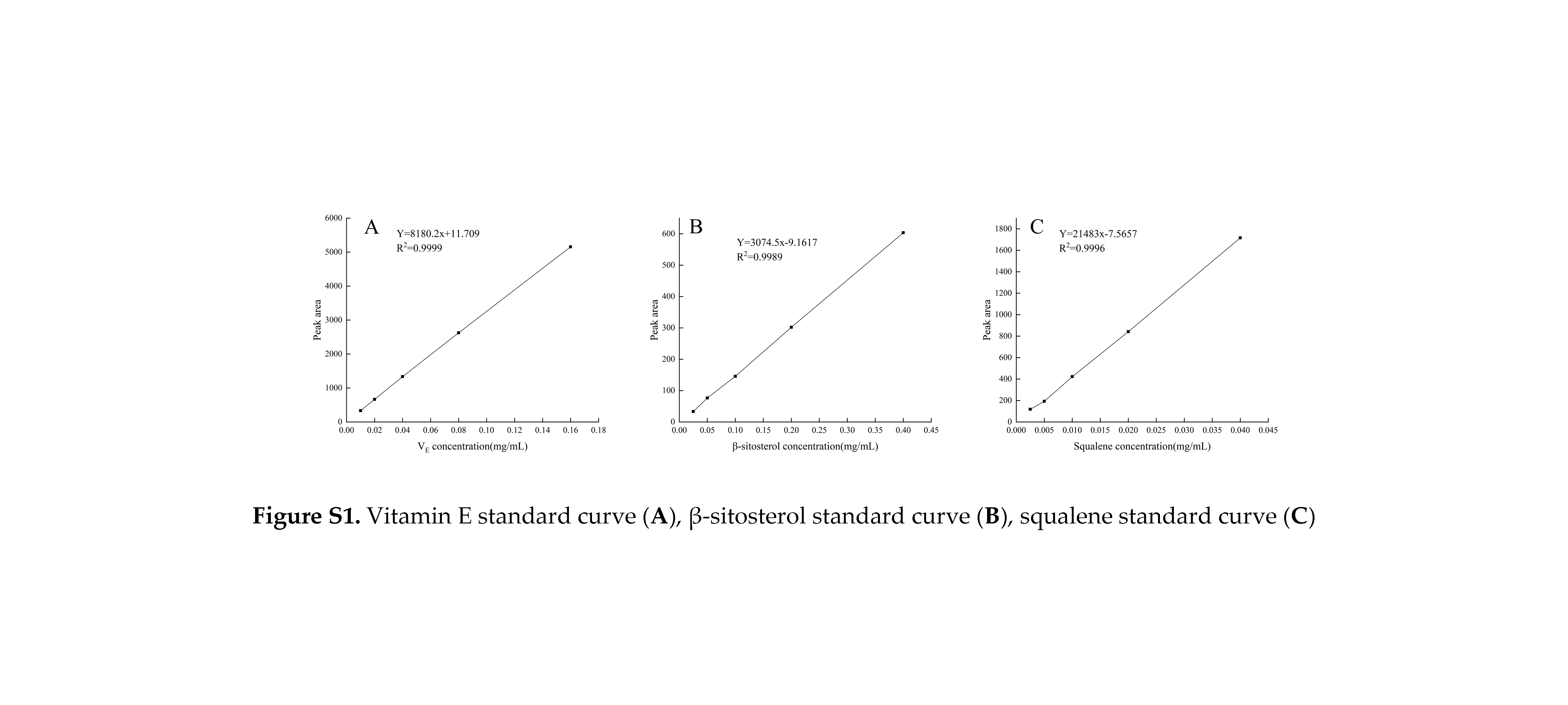

Supplement: Supplementary file 1 [file foods-15-01840-s001.zip › Figure S1.tiff]

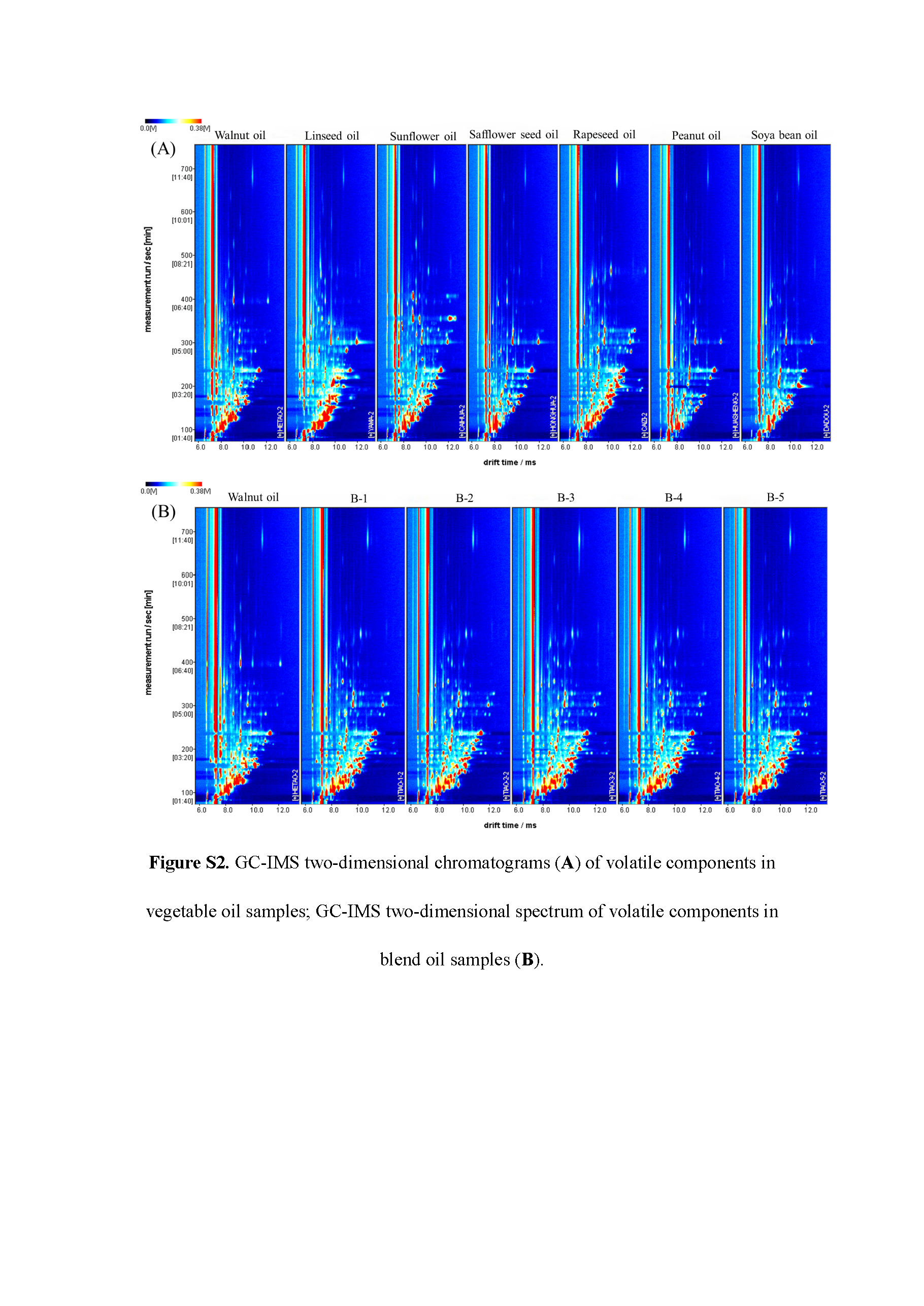

Supplement: Supplementary file 1 [file foods-15-01840-s001.zip › Figure S2.tif]
